# Clinical Safety and Efficacy of Wenxin Keli-Amiodarone Combination on Heart Failure Complicated by Ventricular Arrhythmia: A Systematic Review and Meta-analysis

**DOI:** 10.3389/fphys.2018.00487

**Published:** 2018-05-23

**Authors:** Rui Zheng, Guihua Tian, Qin Zhang, Lin Wu, Yanwei Xing, Hongcai Shang

**Affiliations:** ^1^Key Laboratory of Chinese Internal Medicine of Ministry of Education and Beijing, Dongzhimen Hospital, Beijing University of Chinese Medicine, Beijing, China; ^2^Beijing University of Chinese Medicine, Beijing, China; ^3^Chinese Cochrane Center, West China Hospital, Sichuan University, Chengdu, Sichuan, China; ^4^Department of Cardiology, Peking University First Hospital, Beijing, China; ^5^Guang'anmen Hospital, Chinese Academy of Chinese Medical Sciences, Beijing, China; ^6^Institute of Integration of Traditional Chinese and Western Medicine, Guangzhou Medical University, Guangzhou, China

**Keywords:** Wenxin Keli, amiodarone, heart failure complicated with ventricular arrhythmia, adverse reaction, systematic review, meta-analysis

## Abstract

**Objectives:** To evaluate possible adverse effects and efficacy of Wenxin keli (WXKL)-amiodarone combination on heart failure complicated by ventricular arrhythmia.

**Methods:** Nine electronic literature databases (the Cochrane Library, PubMed, EMBASE, IPA, AMED, CBM, CNKI, VIP, and WanFang) were searched up to February 2018. Two authors extracted data and assessed risk of bias of the included studies independently. Randomized controlled trials (RCTs) and quasi-RCTs about WXKL-amiodarone combination and amiodarone alone were eligible for comparison.

**Results:** Thirteen trials involving 1,126 patients were included. Risk of bias was assessed as high in three studies and unclear in the remaining 10 studies. Six trials reported adverse events (AE). There was no obvious difference between WXKL-amiodarone combination group and amiodarone group in reported AEs (OR 0.64; 95%CI 0.39–1.07). The total effective rate of WXKL-amiodarone combination group was greater than that of amiodarone group (RR 1.22; 95%CI 1.16–1.29). The pooled results showed that the combination group was more effective in reducing heart rate (MD −2.25; 95%CI −2.61 to −1.88, *P* = 0.46, *I*^2^ = 0%), the frequency of ventricular premature complexes (MD −2.03; 95%CI −2.41 to −1.65) and QT dispersion (MD 5.59; 95%CI 3.60–7.58).

**Conclusion:** The WXKL-amiodarone combination is safe and shows more protective effects on heart failure combined with ventricular arrhythmia compared with amiodarone alone. Further research is warranted, ideally involving large, prospective, rigorous trials, in order to confirm these findings.

## Introduction

Heart failure (HF) is a major public health problem, with more than 23 million people worldwide (Roger, 2013). The prevalence of HF is approximately ≥10% among the aged in developed countries (Ponikowski et al., 2016). Within the America alone, the total medical costs for patients with HF are expected to rise from $20.9 billion in 2012 to $53.1 billion by 2030 (Ziaeian and Fonarow, 2016).

Ventricular arrhythmias (VA) include ventricular premature complexes (VPCs), ventricular tachycardia (VT) and ventricular fibrillation. HF disease progression is related to adaptive processes caused by cardiac fibrosis, hypertrophy, leading to adverse left ventricular remodeling and VA. HF and arrhythmia often appear simultaneously and promote each other to deteriorate (Saxon et al., 2006; Goldberger et al., 2011). The higher frequency of VA was associated with heart function decline (Santangeli and Marchlinski, 2015). The mutual mechanism includes inflammation, oxidative and microRNA regulation (Marfella et al., 2013).

Researches have showed that amiodarone treatment is associated with a substantial risk of cardiac and non-cardiac organ toxicity, including thyroid dysfunction (Danzi and Klein, 2015), skin changes, gastrointestinal discomfort (Jaworski et al., 2014). The major adverse events (AEs) are recurrence of arrhythmias and exacerbation of heart failure (Uchida et al., 2014). These adverse effects limit its widespread and long-term usage for all arrhythmia patients (Singh et al., 1995; Khan et al., 2017). Though there are some other approaches for VA such as pacemaker, cardiac resynchronization, anti-arrhythmic agents are still the mainstay therapy (Sardu et al., 2017b). Some new agents or Chinese medicine may serve as an alternative intervention to improve efficacy with reduced AEs.

Wenxin Keli (WXKL), composed of codonopsis pilosula, rhizoma polygonati, pseudo-ginseng, amber, nardostachys, is the first state-sanctioned Traditional Chinese Medicine-based antiarrhythmic drug (Wang, 2013). Several experimental and clinical researches demonstrated WXKL is useful in improving cardiac function and arrhythmia (Chen et al., 2014; Wang et al., 2016; Li et al., 2017a).

The WXKL-amiodarone combination therapy is commonly used and is in clinical (Chen et al., 2014; Li et al., 2017b). However, the effects of WXKL-amiodarone combination for HFVA (heart failure complicated by ventricular arrhythmia) patients remains to be evaluated. Therefore, we sought to evaluate the clinical safety and efficacy of WXKL-amiodarone combination through a systematic review.

## Materials and methods

### Search strategy

This systematic review was conducted according to the Preferred Reporting Items for Systematic Reviews and Meta- analyses: The PRISMA Statement (Moher et al., 2010).

The following electronic databases were searched from date of inception to February 2018: PubMed, EMBASE, AMED (Allied and Complementary Medicine), IPA (International Pharmaceutical Abstracts), the Cochrane Central Register of Controlled Trials (CENTRAL) in the Cochrane Library, China National Knowledge Infrastructure (CNKI), VIP Database, SinoMed Database, and Wanfang Database. The following search terms were used: (“WENXIN KELI” OR “WENXINKELI” OR “wenxin-keli”) and (“amiodarone”) and (“heart failure complicated by ventricular arrhythmia” OR “cardiac failure complicated by ventricular arrhythmia” OR “heart decompensation complicated by ventricular arrhythmia”). We searched for trials from mainstream registries including the World Health Organization International ClinicalTrials Registry Platform (WHO ICTRP; http://apps.who.int/trialsearch/), Current Controlled Trials (http://www.controlled-trials.com), ClinicalTrials.gov trials registry (http://www.clinicaltrials.gov).

### Inclusion and exclusion criteria

Based on the Cochrane Collaboration Handbook standards, the following inclusion criteria were formulated for the selected literatures. The exclusion criteria were as follows: duplicate publications, descriptive studies, animal testing and reports without statistical indicators.

#### Types of studies

Randomized controlled trials (RCTs) or quasi-RCTs were included. No language restrictions, population characteristics and publication types were imposed. We also hand-searched the reference lists of all full text papers for additional relevant reports.

#### Types of participants

Patients with HFVA were eligible to be included. All the participants had to meet at least one of the current or past diagnostic criteria of HFVA, such as the New York Heart Association (NYHA) functional classification and electrocardiogram assessment.

#### Types of interventions

The studied compared WXKL-amiodarone combination with amiodarone, regardless of dosage, type, duration of treatment. The basic therapies in the WXKL-amiodarone combination and amiodarone group were similar.

#### Types of outcomes

The primary outcomes were AEs and total effective rate.

AEs refer to unintended injuries caused by WXKL-amiodarone combination rather than the disease process (World Health Organization, 1966). We classified AEs according to sicken parts and clinical types such as dysfunction. We defined serious adverse events (SAE) as all-cause mortality, severe cardiovascular events, bleeding episode, complication that result in disability caused by therapy.

Secondary outcomes were heart function assessed by the New York Heart Association scale (NYHA), heart rate, the frequency of VPCs, VTs and QT dispersion (QTd).

#### Effective criteria

Remarkable effect: Based on 24 h electrocardiogram, ventricular premature VPCs or VTs disappeared or decreased by 90% or more; NYHA's heart function was up to grade I/II and there was an obvious improvement in the clinical symptom. Effect: VPCs or VTs decreased by 50–90%; NYHA's heart function was up to grade II and the clinical symptom improved partly. Non-effect: It didn't reach the standard of efficiency, and even exacerbation. The total effect = remarkable effect + effect.

### Data extraction and risk of bias assessment

Two authors (Z R, Z Q) independently identified articles for eligibility, and disputes were resolved by discussion with the corresponding author (HC S). Two authors extracted data independently including patient characteristics, details of the combination and amiodarone group, outcome measures and main results. The Cochrane risk of bias tool was used to reduce the risk of bias (Higgins and Altman, 2008), including random sequence generation, allocation concealment, blinding of participants and personnel, blinding of outcome assessment, incomplete outcome data, selective reporting, and other bias.

### Statistical analysis

Statistical analyses were performed in RevMan 5.3 software. Dichotomous outcomes were used pooled risk ratio (RR) with 95% confidence interval (CI) to estimate report effect. Continuous data were presented as mean difference (MD) with 95% CI. Heterogeneity was assessed using the I-squared statistic and I-squared value >50% was considered to be indicative of substantial heterogeneity. The fixed-effects model was used to combine dichotomous data if homogeneity was found. The random-effects model was used if heterogeneity was found.

## Results

### Studies identified

**Figure 1:**
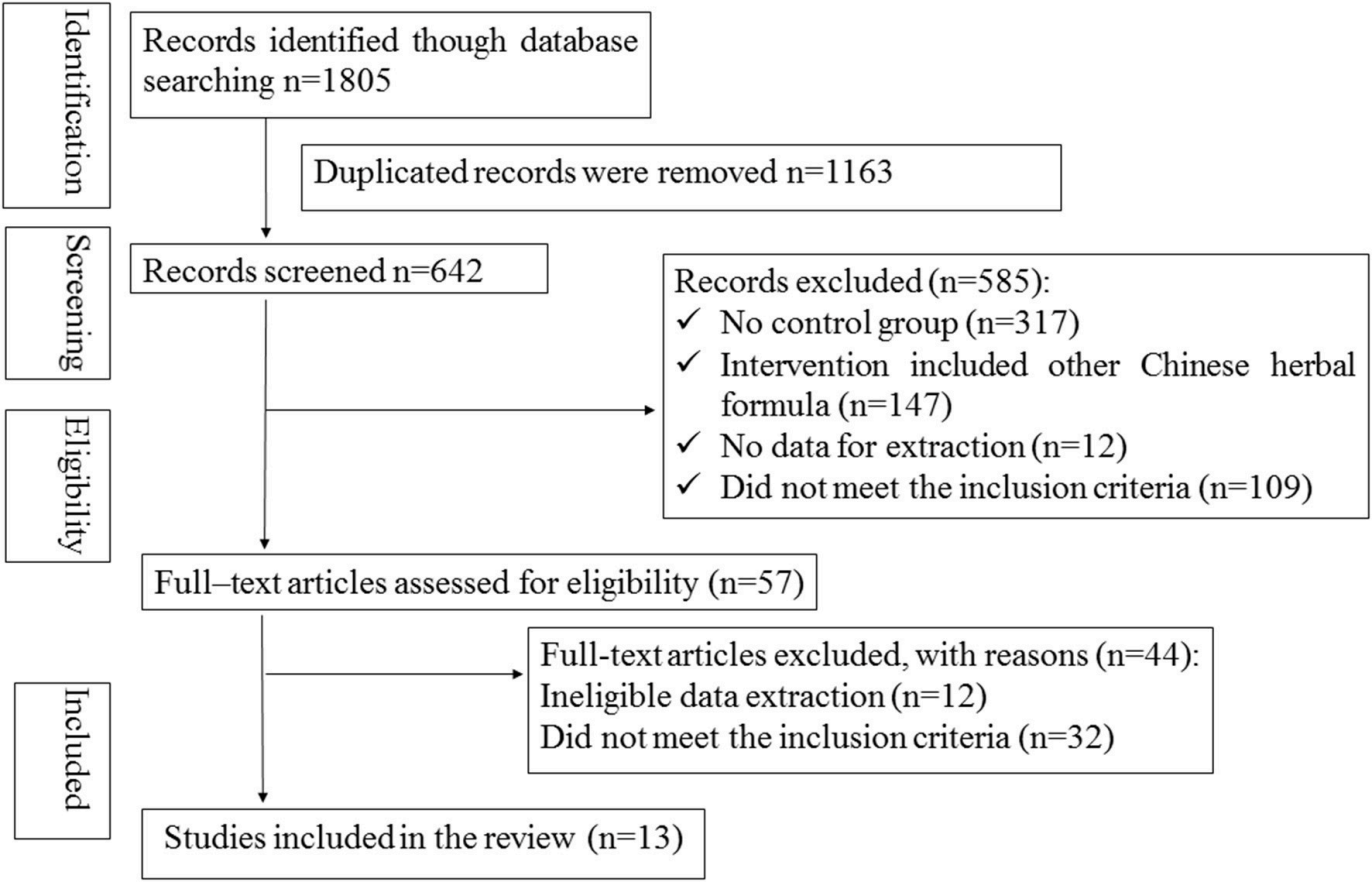
The Preferred Reporting Items for Systematic Reviews and Meta-Analyses (PRISMA) flow diagram.

There were 1,805 potentially relevant references. Thirteen RCTs (World Health Organization, 1966; Wang, 2006; Wang and Song, 2006; Wei et al., 2007; Zhang and Guan, 2007; Shi, 2011; Zhao and Zheng, 2012; Wang et al., 2013; Yan, 2014; Zhou et al., 2014; Wu, 2015; Liu and Rena, 2016; Liu, 2017) were finally included in the meta-analysis. All the RCTs were conducted in China and published in Chinese. Figure 1 showed the search process and study selection. The characteristics of included trials were listed in Table 1.

**Table 1:** Characteristics of included studies.

**Study ID**	**Sample size (Age)**		**Indications**	**Intervention**		**Course (weeks)**	**Outcomes**
	**Combination**	**Amiodarone**		**Combination**	**Amiodarone**		
Zhou et al., 2014	30 (53.5 ± 19.8)	30 (53.5 ± 19.8)	HFVA	WXKL (9 g * Tid)+amiodarone	Amiodarone (0.2 g * Tid * 7 days, maintenance dose 0.2 g*Qd)	4	TER,AR
Zhang and Guan, 2007	30 (Unclear)	30 (Unclear)	HFVA	WXKL (9 g * Tid)+amiodarone	Amiodarone (0.2 g * Tid * 7 days, maintenance dose 0.2 g *Qd)	4	TER
Shen, 2013	62 (61 ± 11.6)	62 (60 ± 10.2)	HFVA	WXKL (18 g * Tid)+amiodarone	Amiodarone (0.2 g * Tid * 7 days, 0.2 g * Bid * next 7 days, maintenance dose 0.2 g *Qd)	Unclear	TER
Zhao and Zheng, 2012	43 (55.6)	43 (52.2)	HFVA	WXKL (9 g * Tid)+amiodarone	Amiodarone (0.2 g * Bid * 28 days)	4	TER, NYHAS
Yan, 2014	41 (52.5 ± 1.7)	41 (52.7 ± 1.6)	HFVA	WXKL (9 g * Tid)+amiodarone	Amiodarone (0.2 g * Bid * 28 days)	4	TER
Wang, 2006	39 (74 ± 7)	31 (71 ± 7)	HFVA	WXKL (9 g * Tid)+amiodarone	Amiodarone (0.2 g * Tid * 7 days, 0.2 g * Bid * next 7 days, maintenance dose 0.2 g *Qd)	4	TER, AR, NYHAS
Liu and Rena, 2016	47 (55.84 ± 6.41)	44 (55.84 ± 6.41)	HFVA	WXKL (9 g * Tid)+amiodarone	Amiodarone (0.1-0.2 g * Tid * 14 days, 0.1 g * Bid * next 14 days)	4	TER, AR, QTd, HR
Wei et al., 2007	86 (58.3 ± 5.2)	86 (58.3 ± 5.2)	HFVPC	WXKL (9 g * Tid)+amiodarone	Amiodarone (0.2 g * Tid * 7 days, 0.2 g * Bid * next 7 days, 0.2 g *Qd* final 14 days)	4	TER, the frequency of VPCs, AR
Shi, 2011	38 (49.55 ± 7.70)	35 (48.00 ± 7. 15)	HFVPC	WXKL (9 g * Tid)+amiodarone	Amiodarone (0.2 g * Tid * 7 days, 0.2 g * Bid * next 7 days, maintenance dose 0.2 g *Qd)	6	TER, the frequency of VPCs
Wang and Song, 2006	39 (54.7 ± 4.4)	39 (54.7 ± 4.4)	HFVPC	WXKL (9 g * Tid)+amiodarone	Amiodarone (0.2 g * Tid * 7 days, 0.2 g * Bid * next 7 days, 0.1–0.2 g *Qd* final 14 days)	4	TER, the frequency of VPCs, AR
Wang et al., 2013	40 (70.2 ± 5.6)	40 (71.6 ± 4.8)	HFVPC	WXKL (9 g * Tid)+amiodarone	Amiodarone (0.2 g * Tid * 7 days, 0.2 g * Bid * next 7 days, 0.2 g *Qd* final 14 days)	4	TER, AR
Wu, 2015	50 (66.5 ± 12.5)	50 (64.4 ± 11.3)	HFVA	WXKL (9 g * Tid)+amiodarone	Amiodarone (0.2 g * Tid * 7 days, 0.2 g * Bid * next 7 days, maintenance dose 0.2 g *Qd)	12	TER, HR
Liu, 2017	25 (55.84 ± 6.41)	25 (55.84 ± 6.41)	HFVA	WXKL (9 g * Tid)+amiodarone	Amiodarone (0.2 g * Tid * 7 days, 0.2 g * Bid * next 7 days, maintenance dose 0.2 g *Qd)	12	TER

*AR, adverse reaction; Bid, two times a day; CE, clinical efficacy; HFVA, heart failure and ventricular arrhythmia; HFVPC, heart failure and ventricular premature complexes; HR, heart rate; NYHAS, New York Heart Association scale; Qd, one time a day; TER, total effective rate; Tid, three times a day; WXKL, WenXin KeLi*.

### Study characteristics

There were 1,126 participants included (570 in the WXKL- amiodarone combination group and 556 in the amiodarone group). Sample sizes of the included studies ranged from 25 to 86. The duration of combination ranged from 4 to 6 weeks. The dosage of amiodarone was 0.2 g three times a day for 7–14 days, 0.2 g two times a day for next 7–14 days, 0.1–0.2 g one times a day for final 7 days.

### Quality of the included studies

**Figure 2:**
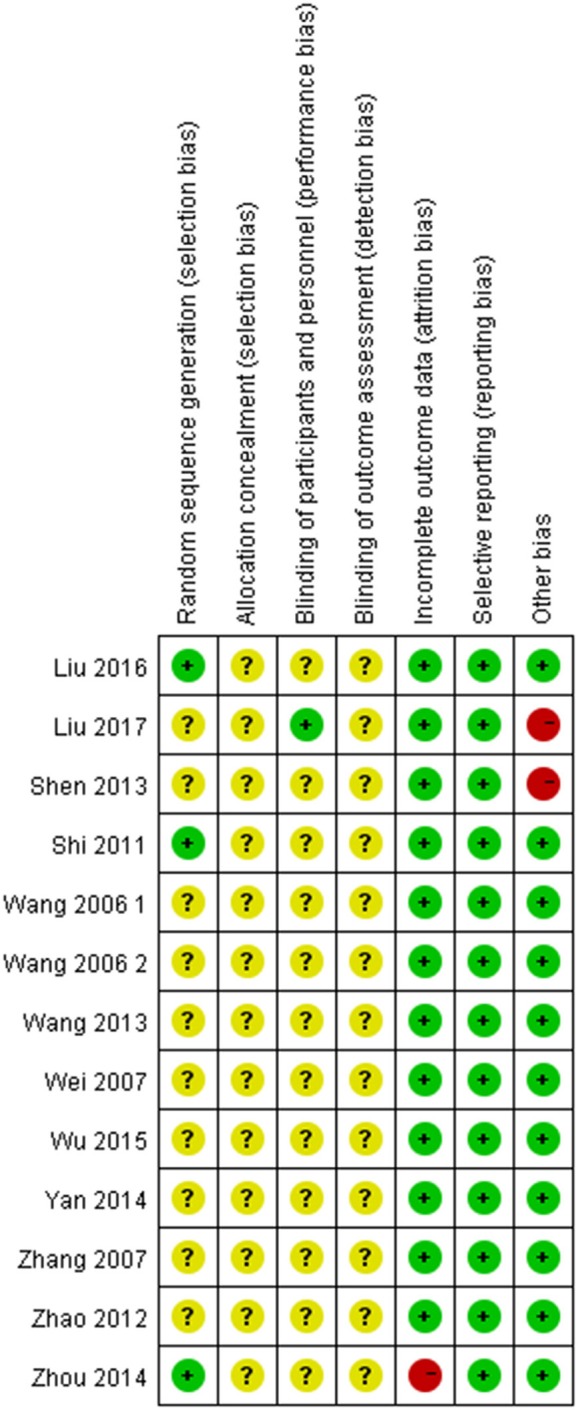
Risk of bias assessment for each included study.

**Figure 3:**
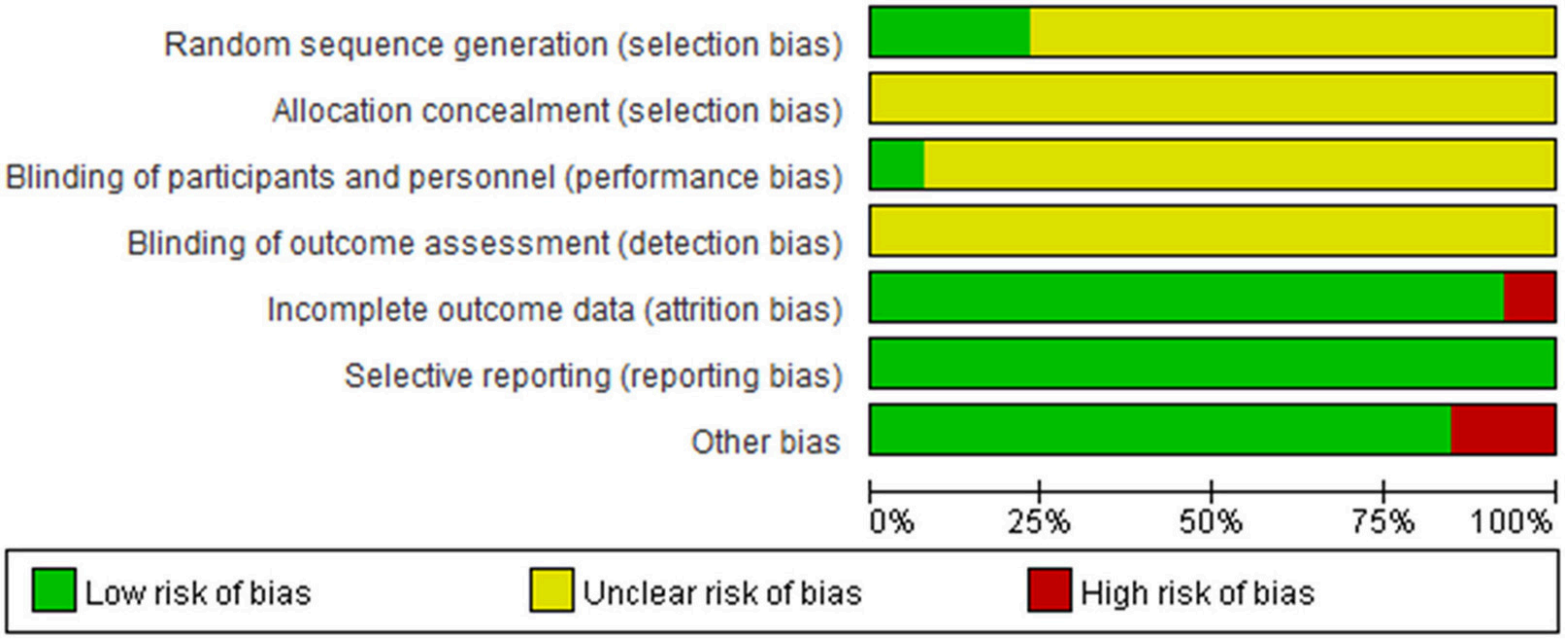
Risk of bias graph.

Randomized allocation of participants was mentioned in all trials. However, only three trials (Shi, 2011; Zhou et al., 2014; Liu and Rena, 2016) claimed that they had used the random number table. One study described dropout and withdrawal data but without ITT (intent-to-treat) analysis (Zhou et al., 2014). One trial didn't report the treatment course (Shen, 2013). One trial report incomplete data (Liu, 2017). In addition, all the trials did not mention allocation concealment, blinding of participants, personnel and outcome assessment. Therefore, the risk of bias of included studies was high, indicating the lack of power to ensure the therapeutic effect. More details of the trials were presented in Figures 2, 3.

### Outcome measures

#### Adverse events

Six studies (Wang, 2006; Wang and Song, 2006; Wei et al., 2007; Wang et al., 2013; Zhou et al., 2014; Liu and Rena, 2016) mentioned AEs and provided an overall sample of 559 patients (285 in the WXKL-amiodarone combination group and 274 in the amiodarone group). Only one study reported no AE in both groups (Wang, 2006). All studies showed no patients suffering from mortality, disability and bleeding episode. AEs in the combination group mainly included severe cardiovascular events (sinus arrest 0.53%), bradyarrhythmia (1.58%), and gastrointestinal disorders (2.99%). AEs in the amiodarone group included severe cardiovascular events (sinus arrest 1.26%), gastrointestinal disorders (9.53%) and dysfunction (0.9%).

**Figure 4:**
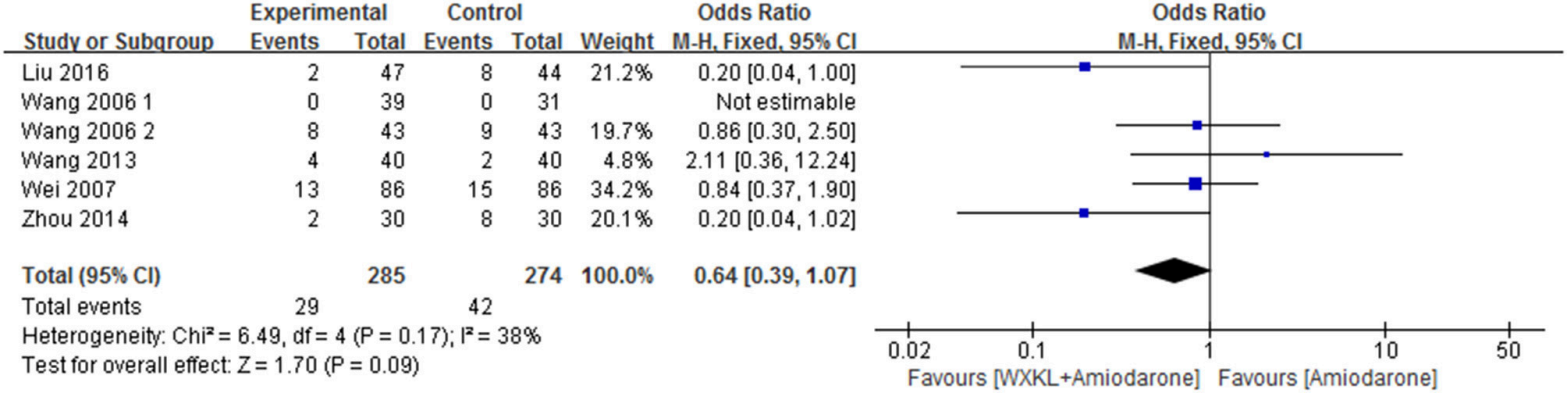
Meta-analysis of the effect of WXKL-amiodarone combination vs. amiodarone alone on adverse reactions.

Meta-analysis of the fixed effects model indicated that there was no significant difference in AEs between WXKL-amiodarone combination group and amiodarone group (OR = 0.64, 95%CI: 0.39 to 1.07, *P* = 0.17, *I*^2^ = 38%, Figure 4). Details were described in Tables 2, 3.

**Table 2:** Type of SARs, involved systems and main clinical main stations.

	**Number of WXKL-amiodarone combination (%^a^)**	**Number of amiodarone (%^a^)**
**SEVERE CARDIOVASCULAR EVENTS**		
Sinus arrest	3 (0.53%)	7 (1.26%)
All-cause mortality	0 (0%)	0 (0%)
Additional major bleeding episode	0 (0%)	0 (0%)
Disability	0 (0%)	0 (0%)

a *The percentage was calculated by dividing the total number of each group's participants by the total number cases of adverse events*.

**Table 3:** Type of ARs, involved systems and main clinical main stations.

	**Number of WXKL-amiodarone combination (%^a^)**	**Number of amiodarone (%^a^)**
**CARDIOVASCULAR EVENTS**		
Bradyarrhythmia	9 (1.58%)	12 (2.16%)
**GASTROINTESTINAL DISORDERS**		
Abdominal discomfort, nausea and vomiting	15 (2.63%)	13 (2.34%)
Gastrointestinal reaction	1 (0.18%)	4 (7.19%)
Bad appetite	1 (0.18%)	0 (0%)
**DYSFUNCTION**		
Hepatic dysfunction	0	3 (0.54%)
Thyroid dysfunction	0	2 (0.36%)

a *The percentage was calculated by dividing the total number of each group's participants by the total number cases of adverse events*.

#### Total effective rate

**Figure 5:**
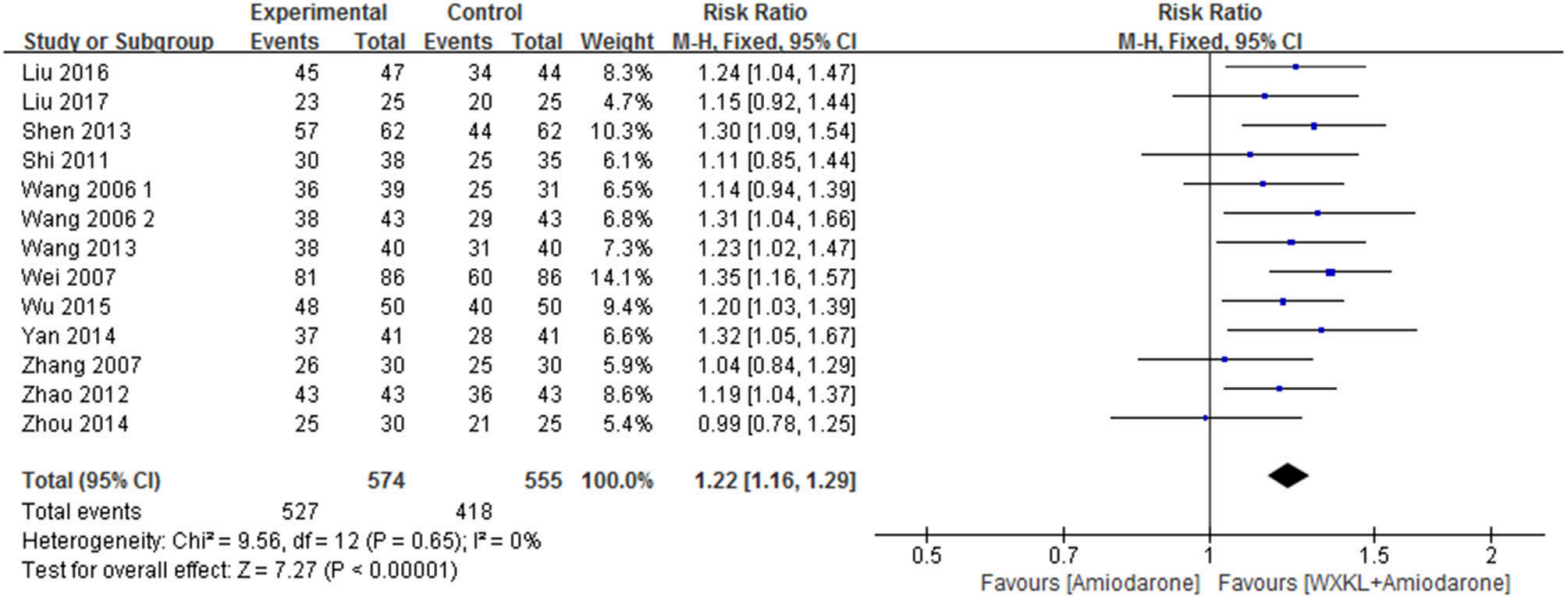
Meta-analysis of the effect of WXKL-amiodarone combination vs. amiodarone alone on the total effective rate.

Thirteen studies contributed to this analysis and involved an overall sample of 1,129 patients (574 in the combination group and 555 in the amiodarone group) (Wang, 2006; Wang and Song, 2006; Wei et al., 2007; Zhang and Guan, 2007; Shi, 2011; Zhao and Zheng, 2012; Shen, 2013; Wang et al., 2013; Yan, 2014; Zhou et al., 2014; Wu, 2015; Liu and Rena, 2016; Liu, 2017). The data were analyzed with a fixed-effects model according to the test of heterogeneity (*p* = 0.65; *I*^2^ = 0%). Over the treatment period, the total effective rate in the WXKL-amiodarone combination group was higher than that in amiodarone group (RR = 1.22, 95%CI 1.16–1.29) (Figure 5).

#### Heart function

**Figure 6:**
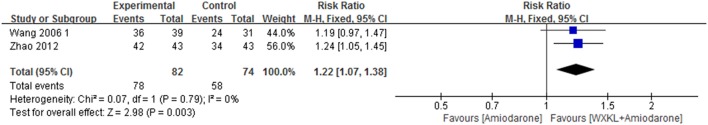
Meta-analysis of the effect of WXKL-amiodarone combination vs. amiodarone alone on heart function.

Only two trials reported the improvement of heart function, evaluated by New York Heart Association scale (Wang, 2006; Zhao and Zheng, 2012). The data were analyzed by using a fixed-effects model according to the test of heterogeneity (*P* = 0.79, *I*^2^ = 0%). Over the treatment period, the heart function was better (RR = 1.22, 95%CI: 1.07–1.38) in the WXKL-amiodarone combination group than amiodarone group (Figure 6).

#### Heart rate

**Figure 7:**
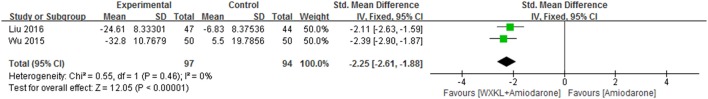
Meta-analysis of the effect of WXKL-amiodarone combination vs. amiodarone alone on heart rate.

There were two studies (Wu, 2015; Liu and Rena, 2016) mentioned the heart rate. Compared with amiodarone alone, the combination group showed significant reduction in heart rate (MD = −2.25, 95%CI: −2.61 to −1.88; *p* = 0.46, *I*^2^ = 0%). As was shown in Figure 7.

#### The frequency of VPCs

**Figure 8:**
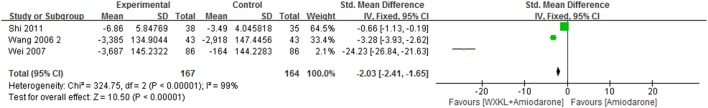
Meta-analysis of the effect of WXKL-amiodarone combination vs. amiodarone alone on the frequency of VPCs.

There were three studies reporting the frequency of VPCs which provided an overall sample of 331 patients (Wang and Song, 2006; Wei et al., 2007; Shi, 2011). The data were analyzed by using a random-effects model according to the test of heterogeneity (*P* < 0.00001, *I*^2^ = 99%). The results showed the combination significantly reduced the frequency of VPCs compared with amiodarone therapy (MD = −2.03, 95%CI −2.41 to −1.65). As was shown in Figure 8.

#### The frequency of VTs

There were none study reporting the frequency of VTs.

#### QT dispersion

There was one study (Liu and Rena, 2016) reporting QT dispersion. Compared with amiodarone, the combination group showed a significant reduction in QT dispersion (MD = 5.59, 95%CI: 3.60–7.58).

## Discussion

This review included 13 studies that involved 1,129 participants. Compared with amiodarone, the combination group showed no significant increase in the AEs (OR = 0.64, 95%CI: 0.39–1.07) and played an advantage in improvement in total effective rate (RR 1.22, 95%CI 1.16–1.29) and heart function (RR 1.22, 95%CI 1.07–1.38). The combination therapy played an advantage in reducing heart rate (MD −2.11, 95%CI −2.63 to −1.59), the frequency of VPCs (MD = −2.03, 95%CI: −2.41 to – 1.65) and QTd (MD = 5.59, 95%CI: 3.60–7.58) compared with amiodarone alone.

Several experimental and clinical researches demonstrated WXKL was useful in reducing heart rate and the frequency of VPCs (Chen et al., 2014; Zhang et al., 2014; Liu, 2017). The antiarrhythmic effect of WXKL benefits from selective inhibition of late sodium current (Hou et al., 2016), L-type calcium current and transient outward potassium current (Chen et al., 2013; Wang et al., 2013; Li et al., 2017b).

A metabolic pro-oxidative and pro-inflammatory status may present in subjects with pro-arrhythmic status, leading to ventricular arrhythmias and worse prognosis (Sardu et al., 2017a). A quantity of experiments confirmed WXKL downregulated genes associated with inflammation, apoptosis (Zheng et al., 2016). One study showed that WXKL ameliorated glucose oxidation degradation to overcome the oxidative stress and the shortage of energy sources in myocardial injury by metabolomics technology (Jiang et al., 2017; Wu et al., 2017b).

The miRNAs may be implicated in adaptive processes such as reverse remodeling during heart failure by regulating cardiac fibrosis, apoptosis, and hypertrophy (Marfella et al., 2013; Sardu et al., 2014, 2016a). WXKL could prevent potential lethal arrhythmia following myocardial infarction by improving gap junctions and miR-1 (Wu et al., 2017a). WXKL regulated neuro-humoral system (ACE and EDN1) and upregulated angiogenesis promoting genes such as RSPO3 (Zheng et al., 2016).

The following advices coming from this research may provide new viewpoints and angles. (1) It is important to explore the pharmacological mechanism of WXKL-amiodarone combination. (2) There must have several methods to gain the follow-up goals. It is important to develop the continuous monitoring systems such as telemedicine to monitor WXKL clinical outcomes and ventricular arrhythmic events (Sardu et al., 2016b). (3) No data have been reported about cardiac resynchronization therapy as an interventional treatment vs. anti-arrhythmic drugs and in association to anti arrhythmic drugs to reduce cardiac arrhythmias, to prevent cardiac arrest, and to improve clinical outcomes (Sardu et al., 2016b).

## Limitations

1. None of the trials had mentioned the follow-up results. AEs of amiodarone-WXKL combination group should be observed and investigated in hereafter follow-up.
2. The details may be the key factors of the AEs. Treatment was not described in details, such as drug interval and others.
3. The risk of bias of included studies was high that it may affect the strength of the results. Most of the trials are small sample studies with positive findings. All included trials were published in Chinese.

A huge number of practitioners in China provide TCM services. Herbal medicines as an adjuvant to conventional therapy should be monitored carefully in the treatment of diseases. There is an obvious need to conduct a lot of full-scale rigorously designed RCTs addressing these limitations.

## Conclusion

In conclusion, the results in this study suggested that WXKL-amiodarone combination group may be as safe as amiodarone alone. The combination played an advantage in improvement in total effective rate and heart function, meanwhile, reduction of heart rate, the frequency of VPCs and QTd. None study reported the frequency of VTs. Additional effective data, well-designed RCTs is needed to prove current findings about the effects of WXKL-amiodarone combination on patients with HFVA. The study in this field is worthwhile and should be continued.

## Author contributions

HS and YX: Defined the research theme; RZ, GT, QZ, and LW: Designed the methods and analyzed the data; RZ, GT, and QZ: Interpreted the results; LW and RZ: Wrote the manuscript; All authors discussed the results and commented on the manuscript.

### Conflict of interest statement

The authors declare that the research was conducted in the absence of any commercial or financial relationships that could be construed as a potential conflict of interest. The handling Editor declared a shared affiliation, though no other collaboration, with one of the authors LW.

## Abbreviations

AE: adverse event
CI: confidence interval
HF: heart failure
HFVPC: heart failure and ventricular premature complexes
HFVA: heart failure complicated by ventricular arrhythmia
HR: heart rate
ITT: intent-to-treat
MD: mean difference
NYHA: New York Heart Association
OR: odds ratio
QTd: QT dispersion
RCTs: randomized controlled trials
RR: risk ratio
SAE: serious adverse event
SMD: standardized mean difference
TER: Total effective rate
VA: ventricular arrhythmias
VPCs: ventricular premature complexes
VTs: ventricular tachycardia
WXKL: Wenxin Keli.
